# The SaeRS Two-Component System of *Staphylococcus aureus*

**DOI:** 10.3390/genes7100081

**Published:** 2016-10-03

**Authors:** Qian Liu, Won-Sik Yeo, Taeok Bae

**Affiliations:** 1 Department of Laboratory Medicine, Ren Ji Hospital, School of Medicine, Shanghai Jiao Tong University, Shanghai 200127, China; qq2005011@163.com; 2 Department of Microbiology and Immunology, Indiana University School of Medicine-Northwest, Gary, IN 46408, USA; wonyeo@iu.edu

**Keywords:** *Staphylococcus aureus*, Two-component system, Virulence factors, Bacterial histidine kinase

## Abstract

In the Gram-positive pathogenic bacterium *Staphylococcus aureus*, the SaeRS two-component system (TCS) plays a major role in controlling the production of over 20 virulence factors including hemolysins, leukocidins, superantigens, surface proteins, and proteases. The SaeRS TCS is composed of the sensor histidine kinase SaeS, response regulator SaeR, and two auxiliary proteins SaeP and SaeQ. Since its discovery in 1994, the *sae* locus has been studied extensively, and its contributions to staphylococcal virulence and pathogenesis have been well documented and understood; however, the molecular mechanism by which the SaeRS TCS receives and processes cognate signals is not. In this article, therefore, we review the literature focusing on the signaling mechanism and its interaction with other global regulators.

## 1. Introduction

The Gram-positive pathogen *Staphylococcus aureus* is a major cause of morbidity and mortality. This bacterium causes a variety of diseases ranging from soft-tissue infections to life-threatening invasive diseases such as endocarditis, toxic shock syndrome, and necrotizing pneumonia [1,2]. The success of *S. aureus* as a human pathogen is largely due to its production of multiple virulence factors, which contribute to various aspects of the bacterial pathogenesis from binding to host tissues to immune evasion [3,4,5]. In *S. aureus*, the production of virulence factors is controlled by an intricate network of transcription regulators including alternative sigma factor σ^B^, DNA binding proteins (e.g., SarA and its homologues) and two-component signaling systems (e.g., AgrAC, ArlRS, SrrAB, and SaeRS) [6,7,8,9,10,11].

The *sae* (*S. aureus* exoprotein expression) locus, which encodes the SaeRS TCS, was identified by Giraudo et al. in 1994 during their characterization of a Tn551 mutant for its defect in the production of exoproteins (e.g., α-hemolysin, β-hemolysin, nuclease, and coagulase) [12,13]. As with other typical TCSs, the signaling cascade in the SaeRS TCS starts when SaeS, the sensor histidine kinase, detects cognate environmental signals (e.g., human neutrophil peptides, HNPs) [14] and autophosphorylates at the conserved His131 residue. The phosphoryl group is then transferred to Asp51 of SaeR, and the phosphorylated SaeR (SaeR-P) binds to SaeR binding sequence (SBS) and, in most cases, activates the transcription of the target genes. Due to its profound effects on staphylococcal virulence gene expression and pathogenesis, the SaeRS TCS (Sae system) has been a target of extensive research, and the roles of the TCS in virulence gene expression and staphylococcal pathogenesis are well documented and understood [15,16,17]. Therefore, in this article, we will review the literature focusing on the molecular mechanism of cell signaling. In addition, at the end, we will briefly discuss the interaction of the SaeRS TCS with other regulatory systems and its role in biofilm formation and staphylococcal virulence.

## 2. Components of the SaeRS TCS

### 2.1. Structure of the *sae* Operon

**Figure 1:**
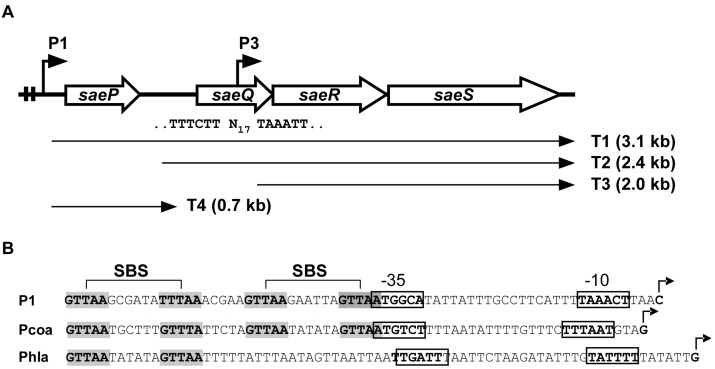
The *sae* operon. (**A**) Organization of the *sae* operon. Two angled arrows represent the P1 and P3 promoters, respectively. Two vertical lines in the P1 promoter region indicate the SaeR binding sequences (SBSs). The nucleotide sequence of the P3 promoter is shown under *saeQ,* where N_17_ = 17 nucleotides. Four transcripts (T1–T4) are indicated by arrows; (**B**) DNA sequences of select *sae* target promoters. The SBSs are shown in gray. Transcription start sites are indicated by angled arrows. The promoter sequences are shown in boxes. Pcoa, the promoter of coagulase (*coa*); Phla, the promoter of α-hemolysin (*hla*).

The *sae* operon consists of four genes (*saeP, saeQ, saeR*, and *saeS*), and the two promoters P1 and P3 (Figure 1) [15,18,19].

#### 2.1.1. P3 Promoter (*sae*P3)

The P3 promoter is located inside *saeQ* and transcribes only *saeR* and *saeS* (i.e., the T3 transcript in Figure 1A) [15,18]. The P3 promoter is weaker than P1 and constitutive [18,20]. The transcription of *saeRS* from P3 is sufficient for activation of the Sae target genes. In fact, deletion of the *sae* sequence upstream of P3 did not significantly affect exoprotein production [18]. Therefore, the constitutive P3 promoter provides the basal levels of SaeS and SaeR for sensing and responding to cognate signals.

#### 2.1.2. P1 Promoter (*sae*P1)

The P1 promoter resides in the front of the very first gene *saeP* and can transcribe all four genes (Figure 1A). As compared with P3, P1 is much stronger and, due to two SBSs, it is autoinduced by the SaeRS TCS (Figure 1B) [15,19]. From the P1 promoter, T1 transcript is produced and further processed into T2 and T4 (Figure 1A). When P1 is induced, due to the increased transcripts, the SaeRS protein levels also increase. However, the increase of SaeRS is not expected to further increase the activity of the SaeRS TCS because (1) overexpression of *saeRS* does not alter the expression pattern of the Sae*-*regulon (*coa*, *eap*, and *hla*) [21], and (2) the production of SaePQ from P1 reduces the overall Sae activity by inducing SaeS’s phosphatase activity (see SaeP & SaeQ below) [18].

### 2.2. SaeS

**Figure 2:**
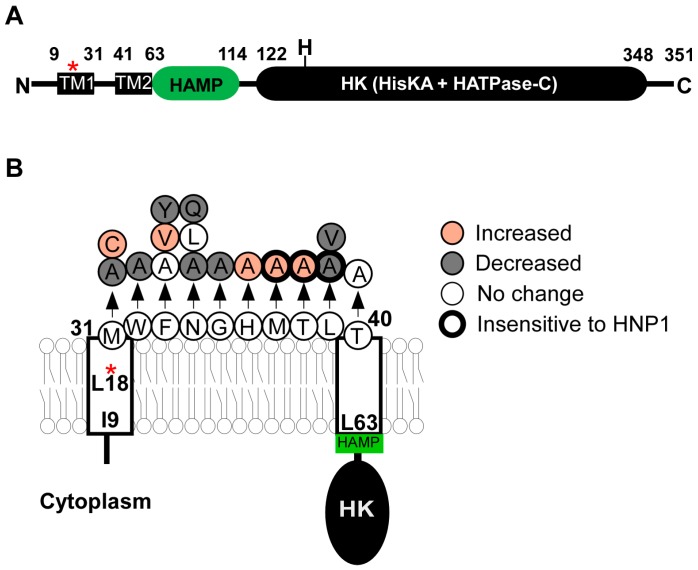
The SaeS protein. (**A**) The domain structure of SaeS. The numbers represent the boundary amino acids. The red star indicates the L18P mutation of SaeS in the strain Newman. N,N-terminus; H, His 131; C,C-terminus; TM, transmembrane helix; HK, histidine kinase; (**B**) A summary of mutations in the transmembrane domain of SaeS. Increased, Increased basal kinase activity; Decreased, Decreased basal kinase activity; No change, No effect on the basal kinase activity; Insensitive to HNP1, Kinase activity does not respond to HNP1. The positions of amino acids are all predictions.

SaeS is composed of 351 aa where His131 is the phosphorylation site [13]. As a sensor histidine kinase, its kinase activity determines the level of SaeR-P, the effector molecule of the SaeRS TCS. Although, as a bifunctional histidine kinase, SaeS is supposed to have both kinase and phosphatase activities, purified MBP (maltose binding protein)-SaeS fusion protein shows no significant phosphatase activity [22]. On the other hand, SaeS has a low but significant basal kinase activity, which allows the expression of *hla* without induction (see Section 4 for detailed discussion) [21,23]. SaeS consists of a transmembrane domain, a HAMP domain, and a kinase domain (Figure 2A). Although the exact boundary of transmembrane domain has not been experimentally determined, SaeS is predicted to have a nine aa-long linker peptide between two transmembrane helices [23,24]. Since the linker peptide is too small to serve as a ligand binding domain, SaeS is classified as a member of intramembrane-sensing HKs (IM-HK) [25,26].

#### 2.2.1. Transmembrane Domain

In SaeS, the transmembrane domain of SaeS is necessary and sufficient to respond to the activation signal human neutrophil peptide 1 (HNP1) [23]. Although SaeS of *Staphylococcus epidermidis* does not respond to HNP1, when the transmembrane domain was swapped with that of *S. aureus*, the hybrid SaeS did [23]. In addition, the transmembrane domain affects the basal kinase activity of SaeS. For example, in the strain Newman, the L18P mutation in the first transmembrane helix dramatically increases the kinase activity of SaeS [24,27]. Alanine substitutions of the linker peptide also altered the kinase activity of SaeS and/or the response to HNP1 (Figure 2B) [23,28]. Two hypotheses were proposed to explain how the transmembrane domain affects the kinase activity of SaeS. Flack et al. [28] found that the amino acids Met, Trp, and Phe (aa 31–33, MWF in Figure 2B) are conserved in all SaeS orthologues in staphylococci, and that the alanine substitutions of those amino acids, in particular, the Met31 residue, greatly reduced α-hemolysin (*hla*) promoter activity and hemolytic activity of culture supernatant. Based on those results, Flack et al. proposed that the conserved amino acids are critical for SaeS kinase activity. On the other hand, Liu et al. [23] found that the effects of amino acid substitutions in the linker peptide vary depending on the substituting amino acids. For example, although the M31A substitution decreased the kinase activity of SaeS, the M31C substitution increased it. Similarly, the F33Y substitution decreased the kinase activity of SaeS whereas the F33V substitution increased it (Figure 2B). Based on these results, Liu et al. proposed that the overall conformation of the transmembrane domain, not MWF residues, is critical for the SaeS’s kinase activity (a tripwire model) [23]. This model predicts that any molecules altering the conformation of the transmembrane domain can serve as a signal for SaeS.

#### 2.2.2. Growth-Phase Dependent Activation

The transcription of the *sae* operon from the P1 promoter is affected by growth phase and is maximal in the post-exponential growth phase [15,19]. The transcription of the Sae target genes such as *fnbA,* *coa*, and *hla* showed growth-dependency even in the mutant of Agr, the staphylococcal quorum sensing system, indicating that the growth-phase-dependent Sae activation is independent of Agr [29,30]. The molecular mechanism underlying the results is unknown. However, since the kinase activity of SaeS determines the extent of the Sae target gene activation, these results suggest that the kinase activity of SaeS increases in a growth dependent but Agr-independent manner.

### 2.3. SaeS Variants

SaeS shows polymorphisms, and three SaeS variants are known: SaeS^P^, SaeS^SK^, and SaeS^SKT^.

#### 2.3.1. SaeS^P^ (SaeS L18P)

This SaeS variant has a L18P substitution mutation (T53C mutation in *saeS*) in the first transmembrane helix (Figure 2). This variant protein was originally identified in the strain Newman [19,24], but is also found in other strains too (e.g., LysK 1 2010, Lyso 1 2010, 1101-2 2010, 1801-1 2010) [117]. Due to the substitution mutation, the kinase activity of SaeS^P^ is highly increased. For example, the cell lysate of the strain USA300, which produces wild type SaeS (SaeS^L^), did not visibly phosphorylate SaeR; however, the Newman cell lysate did [31]. Along with the heightened kinase activity, signal response was also altered in SaeS^P^. When treated with 0.006% SDS (sodium dodecyl sulfate), the kinase activity of SaeS^P^ was further increased whereas that of wild type SaeS was inhibited [27]. Since other detergents such as Triton X-100 and Tween 20 did not affect the kinase activity of SaeS^P^ [27], SDS seems to be a specific signal for SaeS^P^. Due to the high kinase activity of SaeS^P^, the auxiliary proteins SaeP and SaeQ are constantly expressed in the strain Newman. However, those auxiliary proteins are not involved in the SDS-mediated activation of SaeS^P^ [32]. Co-expression of SaeS^L^ suppressed the target gene expression by SaeS^P^ [21,24], indicating that SaeS^P^ can have high kinase activity only in a form of homodimer.

Another unique trait of SaeS^P^ is its instability in the absence of SaeQ. Without SaeQ, almost no SaeS^P^ was detected [18]. This result indicates that SaeS^P^ is subject to proteolytic degradation in the membrane, from which SaeQ protects SaeS^P^.

#### 2.3.2. SaeS^SK^ and SaeS^SKT^

SaeS^SK^ has two substitution mutations (N227S and E268K) and is found in the strains MW2, Mu50 and USA600 [33]. Those substitutions, however, do not seem to alter the enzymatic activity of SaeS because they do not affect the production of nuclease, a member of Sae-regulon [33]. Recently, SaeS^SKT^, a SaeS^SK^ variant with one more amino acid substitution at the last position (S351T) was reported in ST30 and ST36 lineages of *S. aureus* [34]. Interestingly, unlike the wild type Sae system, whose activity increases in a growth phase-dependent manner and is maximal at the post-exponential growth phase [15,19], the activity of the Sae system with SaeS^SKT^ appeared to be highest in the exponential growth phase and reduced in the stationary growth phase [34]. The molecular basis of the altered activation pattern remains to be elucidated.

### 2.4. SaeR

SaeR is an OmpR family response regulator composed of 228 amino acids (pI = 5.2.). At its N-terminus, SaeR contains the receiver domain where Asp51 is the phosphorylation site. Phosphorylation at Asp51 is essential for SaeR to bind its target DNA [31]. Indeed, the D51N substitution in SaeR abolished the expression of *coa* and *hla* [21]. At its C-terminus, SaeR contains the DNA binding domain (SaeR^DB^). Unlike SaeR, which does not bind DNA without phosphorylation, SaeR^DB^ does bind DNA, demonstrating that, in SaeR, the unphosphorylated receiver domain prevents the DNA binding of SaeR^DB^ [31]. The crystal structures of SaeR^DB^ (aa 125–228 or 128–228) revealed that SaeR^DB^ exists as a monomer with a winged helix-turn-helix module [35,36]. From the structural studies, K174, H198, R199, R201, W218 were predicted and confirmed to be critical for DNA binding and transcription activation of target genes [35].

#### SaeR Binding Sequence (SBS)

**Figure 3:**
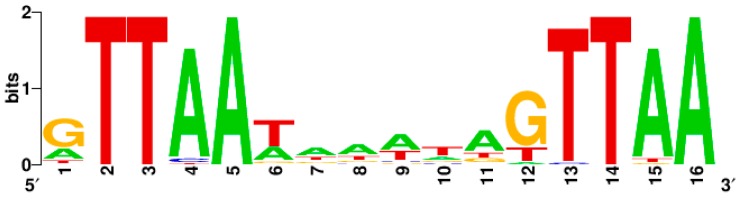
The consensus sequence of the SaeR binding sequence. It was generated by WebLogo (http://weblogo.berkeley.edu/logo.cgi) using the SaeR binding sequences in Table 1.

The perfect binding sequence of SaeR-P is GTTAAN_6_GTTAA, where N = any nucleotide with preference to A/T [31,37] (Table 1). Of the GTTAA sequence, G is not absolutely required for the SaeR-mediated transcription activation while TTAA appears to be. For instance, when G was mutated to C in P1, the mutant P1 still showed significant promoter activity (~20% of wild type); however, when similar single substitution mutations were made in the TTAA sequence, the resulting mutant P1 promoters showed almost no promoter activity [31]. Indeed, the consensus SaeR binding sequence indicates that the TTAA sequence plays a major role in SaeR-mediated transcription activation (Figure 3).

### 2.5. SaeP and SaeQ

**Figure 4:**
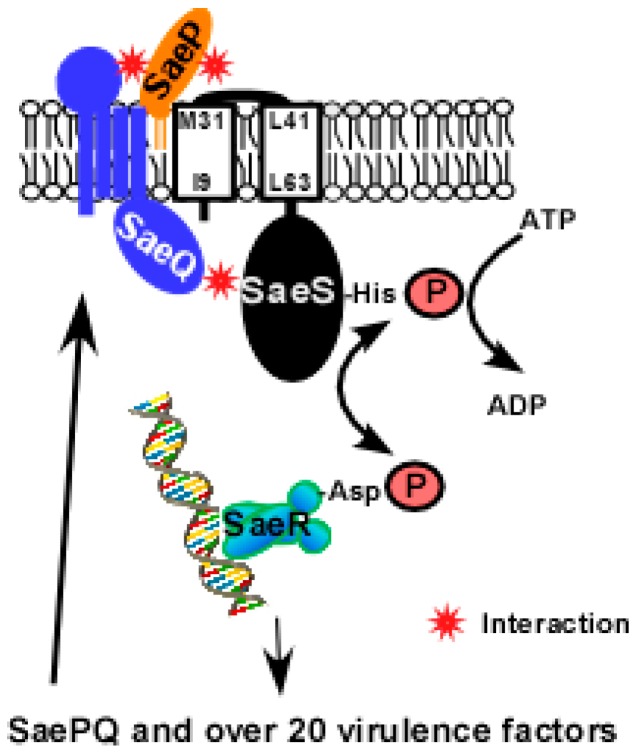
A model for SaePQS interaction. Upon exposure to the inducing signal(s), SaeS autophosphorylates the conserved His residue with ATP; then the phosphoryl group is transferred to the conserved Asp residue of SaeR. The phosphorylated SaeR binds to its binding sequence and activates transcription from target promoters including the *sae*P1 promoter. From *sae*P1, SaeP and SaeQ are produced and bind to SaeS in the membrane. As a lipoprotein, SaeP is expected to interact with the extracellular linker peptide of SaeS. On the other hand, SaeQ is thought to interact with the cytoplasmic domain of SaeS [22]. The interacting parts of SaePQS are based on predictions.

*saeP* and *saeQ* are auxiliary genes upstream of *saeRS* [15]. *saeP* encodes a lipoprotein of 146 aa (pI = 9.87) while *saeQ* encodes a membrane protein of 157 aa (pI = 9.76) with four predicted transmembrane helices (Figure 4) [19,22]. The N-terminal sequence of SaeP is known to show one or more polymorphisms [19]. Neither SaeP nor SaeQ shows significant homology to proteins of known functions. SaePQ are dispensable for the activation of the Sae system [18,21], however, they are required to induce SaeS’s phosphatase activity by forming a SaePQS ternary complex (Figure 4) [22]. Interestingly, the phosphatase activity of the cytoplasmic domain of SaeS (SaeSc), which cannot interact with SaeP, is also enhanced by SaePQ in vitro [22]. Therefore, it is likely that the SaeQ-SaeS interaction in the cytoplasm is directly responsible for the activation of SaeS’s phosphatase activity. The interacting parts of SaeP, SaeQ and SaeS, and the molecular mechanism of the activation are not yet known.

## 3. Signals

### 3.1. Activation Signals

#### 3.1.1. Human Neutrophil Peptide 1, 2, and 3 (HNP1-3)

Human neutrophil peptide 1, 2, and 3 (HNP1-3) are antimicrobial peptides produced by human neutrophils, constituting 30% to 50% of the total protein in azurophilic granules, the primary contributor to the killing and degradation of microorganisms in phagolysosomes [38]. HNP1-3 activate the Sae system at subinhibitory concentrations (0.5–20 µg/mL, MIC = 200 µg/mL) [14]. The activation is specific because other antimicrobial peptides such as LL37, daptomycin, and vancomycin show no effect on the Sae system [14]. Intriguingly, the HNP effect is strain-specific. Although HNP activates the Sae system in the strains USMS-1, MW2, N315, USA300, MRSA252, and MSSA476, it does not in the strains ISP479R, COL, and Newman [14]. The strain-specific effect of HNP has not yet been explained. It is also unknown whether HNPs directly bind to SaeS or not. Since HNPs are produced by human neutrophils, it is not surprising that human neutrophils also activate the Sae system [39,40]. Intriguingly, the Sae system is also activated by murine neutrophils, which do not produce alpha-defensins like HNP1-3 [40,41,42], indicating that other Sae-activating molecules are present in murine neutrophils.

#### 3.1.2. Calprotectin

Calprotectin is a member of the EF-hand calcium binding protein family and is composed of the heterodimeric complex of S100A8 /A9 (also called Mrp8/14) [43]. It is abundantly expressed in neutrophils, constituting approximately 50% of neutrophil cytoplasmic proteins, and its concentration in tissue abscesses is over 1 mg/mL [44,45]. In abscesses, calprotectin suppresses staphylococcal growth by sequestering the nutrient metal ions Zn and Mn [45,46]. The Zn-bound form of calprotectin is known to protect the Sae system from inhibition by Zn and Fe [42], although the mechanism is still unknown.

#### 3.1.3. Other Activation Signals

**Figure A1:**
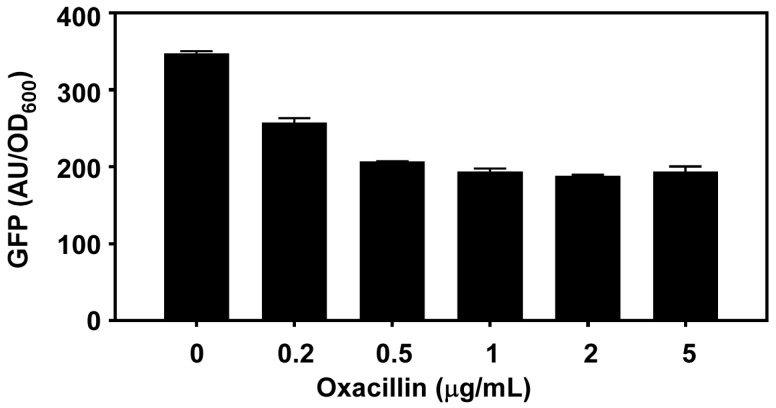
Effect of oxacillin on P1 promoter activity. *Staphylococcus aureus* USA300 carrying P1-GFP reporter plasmid [42] was grown in 150 µL TSB at 37 °C until mid-exponential growth phase. After addition of oxacillin to the concentration indicated, the culture was further incubated for 2 h at 37 °C, and OD_600_ and GFP expression were measured (Enspire, Perkin Elmer, Waltham, MA, USA). AU, arbitrary unit.

Inhibitory concentrations of hydrogen peroxide and subinhibitory concentration of beta-lactam antibiotics were reported to activate the Sae system [14,47,48]. However, in our hands, beta-lactam antibiotics such as oxacillin does not activate the SaeRS TCS but rather reduced the *sae* transcription from the P1 promoter (Appendix Figure A1). Therefore, the effect of beta-lactams is strain-specific, and it is unlikely that beta-lactam antibiotics are signal molecules for the Sae system.

### 3.2. Inhibitory Signals

#### 3.2.1. Silkworm Apolipophorin Protein

This protein is known to reduce the transcription of the hemolysin genes (*hla* and *hlb*), *saeS,* and *saeQ* [49]. It binds to lipoteichoic acid (LTA), and the LTA complexed with the protein inhibits the kinase activity of SaeS by interacting with transmembrane domain of SaeS, in particular, the linker peptide region [50]. When SaeS was mutated in the transmembrane helices (i.e., I9Q and I9Q/L63Q) or in the linker peptide (i.e., Δ34–36, Δ35–37, Δ34–37), the kinase activities of the resulting mutant SaeS proteins were either non-responsive to (I9Q) or activated by the apolipophorin protein [50].

#### 3.2.2. Acidic pH and 1M NaCl

At pH 5.5 or in the presence of 1 M NaCl, the P1 promoter activity was repressed [14,51] although it was not inhibited in the Newman strain background (i.e., SaeS^P^ variant) [24].

#### 3.2.3. Other Potential Sae Inhibitory Molecules

The human skin fatty acid *cis*-6-hexadecenoic acid was reported to repress the Sae system, although the mechanism is not known [52]. 18β-glycyrrhetinic acid, a component of the licorice root *Glycyrrhiza* spp., was also shown to inhibit the transcription of *saeR* and *hla*, raising the possibility that it might specifically repress the Sae system. Schmitt et al. showed that haemin reduces the transcription of *hlb* and *hlgA* in a Sae-dependent manner [53] while Baker et al. showed that Cu represses the Sae system [54]. Indeed, in an in vitro enzyme assay, Cu, along with Zn and Fe, was shown to inhibit the kinase activity of SaeS [42], possibly competing with Mg in SaeS. Finally, the following molecules have been reported to inhibit the expression of the *sae*-operon or some members of Sae-regulon and have a potential to be a Sae inhibitor: florfenicol [55], corilagin [56], licochalcone A [57], thymol [57], the enoyl-acyl carrier protein reductase inhibitor AFN-1252 [58], cerulenin [59], subinhibitory concentration of linezolid [60], Manuka honey [61], and flavone [62].

## 4. The *S*ae Target Genes

The direct Sae target genes harboring SBS in their promoter region are listed in Table 1. Two different classes of the Sae target promoters were identified. The class I (or low affinity) targets (e.g., *coa, fnbA, eap, sbi, efb, fib,* and *saeP*) are highly expressed in strain Newman, which has SaeS^P^, while the class II (or high affinity) targets (*hla* and *hlb*) are insensitive to the SaeS polymorphism [21,23]. The class I target promoters appear to have a low affinity for SaeR-P, and their transcription requires relatively high levels of SaeR-P. On the other hand, the class II target promoters seem to have a high affinity to SaeR-P, and the basal level of SaeR phosphorylation is sufficient for their transcription [21]. Further study on the *coa*, *sae*P1, and *hla* promoters has shed light on the molecular basis of their different SaeR-P requirements. First, the class I targets such as *coa* and *sae*P1 promoters have two SBSs, while the class II target *hla* promoter has only one (Figure 1B). Therefore, regardless of their binding affinities to SaeR-P, the transcription of the class I targets will require twice as much SaeR-P as that for the class II targets. Second, in the absence of SaeR-P, the class I target promoters (i.e., *coa* and *sae*P1) do not bind to RNA polymerase whereas the class II target promoter (i.e., *hla*) does [63], indicating that, even in the absence of SaeR-P, the *hla* promoter can be occupied by RNA polymerase and be ready for transcription.

**Figure 5:**
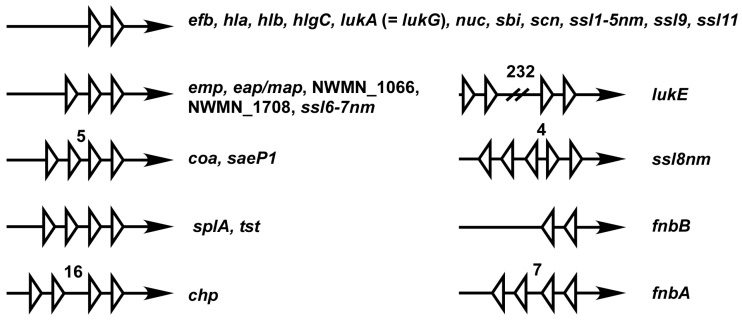
Organization of SBSs in the *sae* target gene promoters. Each white triangle represents a half of the SaeR binding sequence. Arrows represent promoter region pointing the direction of transcription. Unless stated otherwise, the distance between each half binding sequence is 6 bp. Numbers are base pairs of the gap between the half-binding sites.

The organization of SBSs varies among the Sae target promoters (Figure 5). Such variations along with promoter strength are expected to affect the sensitivity of those promoters to SaeR-P and to shape the overall regulation patterns. Of the promoters, six have 1.5 SBS (i.e., three half binding sites). Since it is unlikely that all three half-binding sites are occupied by SaeR-P simultaneously, one of the half-binding sites at the boundary might be non-functional, leaving only one SBS. In the promoters of *ssl8nm*, *fnbA* and *fnbB*, SBSs are placed in an opposite orientation. It remains to be determined how SaeR-P activates transcription from those promoters.

Although the Sae system mostly activates target gene expression, some genes are known to be downregulated by the system. In the strain Newman, a *sae* mutant expressed more capsular antigen than the wild type did [19,75]. Indeed, the *cap* promoter activity was also higher in the *sae* mutant [19]. However, since the putative SBS in the *cap* promoter is rather poorly conserved (Table 1), it is not clear whether or not the SaeR-P directly binds and represses the *cap* promoter. The Sae system is also known to repress the expression of aureolysin (Aur), a zinc metalloproteinase [24,66,74]. In the *sae* mutant, the increased production of Aur contributes to the lower level of exoproteins in the culture supernatant [74]. However, the putative SBS in *aur* is also poorly conserved (Table 1), and the transcription of the *aur* gene was not significantly affected in the *saeS* mutant [66]. Therefore, it is possible that the Sae-mediated repression of *aur* is indirect.

## 5. Interaction with Other Regulators

As shown in Table 1, the Sae system does not directly control the transcription of other global regulatory genes such as *agr*, *sigB*, *sarA*, or *rot* [14,20,64,66,76]. However, the transcription of the *sae*-operon is known to be affected by other regulatory systems.

### 5.1. Agr

Agr is the staphylococcal quorum sensing two-component system where AgrC and AgrA are the sensor histidine kinase and the response regulator, respectively [77]. AgrC is activated by autoinducing peptide, which is encoded by *agrD*, and phosphorylates AgrA. The phosphorylated AgrA (AgrA-P) binds to the P2 and P3 promoters of the *agr* operon and activates the transcription of *agrBDCA* and RNAIII, the major effector molecule of the Agr system. Along with the *agr*P2 and P3 promoters, the *psm* (phenol soluble modulin) promoters are known to be a direct target of AgrA [78].

Based on the effect of an *agr* mutation on the transcription of the *sae* operon, the Sae system has been suggested to be downstream of Agr in the exoprotein production pathway [15]. For example, *agr* mutation reduced both the *sae*P1 promoter activity and the Sae mRNA level [13,14,15,79]. In the strain ISP479, a derivative of the strain 8325-4, an *agr* mutation greatly reduced the *sae*P3 promoter function in the 1.15 kb fragment upstream of *saeR* [80]. Novick et al. also showed that RNAIII was required for *sae* transcription from *sae*P1 [15]. However, other experimental results also suggest that Agr and Sae are independent of one another. First, the *sae* operon contains neither AgrA nor RNAIII binding sequences. Therefore, the effect of the *agr* mutations on the *sae* expression must be indirect. Second, some target genes are regulated by Agr and Sae in an opposite manner. For example, *coa* and *fnbA* are repressed by Agr but activated by Sae [19,64,81,82]. The *cap* gene is activated by Agr but repressed by Sae [19,83,84]. In the case of TSST-1, the toxin responsible for toxic shock syndrome [85], both Agr and Sae are positive regulators [73]. However, the deletion of *agr* did not abolish the production of TSST-1 whereas *sae*-deletion did [73]. Third, several in vivo model experiments showed that Sae is critical for toxin production whereas Agr is dispensable. For instance, during device-related infection in guinea pigs, Sae was required for the expression of *hla* while Agr was not [76], despite the fact that, in the in vitro condition, Agr positively regulates the *hla* expression with RNAIII at a post-transcription level [86]. During murine skin infection of the USA300 strain, the Sae target genes such as *hlgA*, *hlgB*, *hlgC*, and *lukA* were highly expressed in the *agr* mutant but abolished in the *sae* mutant [40]. In an infective endocarditis model, the *hla* transcript level was significantly reduced in the *sae* mutant but not in *agr*, *sarA*, or *agr/sarA* mutants [4]. Steinhuber et al. showed that, under elevated CO_2_, Sae is critical for the hemolytic phenotype but Agr is not [19]. These results cannot be explained by the direct regulation of Sae by Agr. Finally, as stated in Section 2.1, the target gene expression is not affected by the expression level of the *sae* operon [21]. Therefore, even though Agr might affect the *sae* transcription from P1, it would not affect the overall Sae activity (i.e., the expression level of the Sae targets). It should be noted that some results from early studies are questionable. For example, in the 2003 study by Giraudo et al. [80], despite the fact that *agr* mutation abolished the promoter activity of *sae*P3 at OD_600_ = 4, no significant changes were observed in the transcript profile analyzed by Northern blot. Moreover, in most studies, the effect of *agr* mutation on the *sae* expression was not confirmed by a complementation test [13,14,79]. Recently, Baroja et al. showed that, in the strain MN8, the deletion of *agr* did not affect the transcription of *saeR* [73], further confirming the independence of Agr and Sae.

### 5.2. WalRK

In *S. aureus*, the WalRK TCS is essential for cell viability and plays a key role in cell wall metabolism [87,88]. When a constitutively active form of the response regulator WalR (i.e., WalR D55E) was produced, most members of the Sae regulon including the *sae* operon were up-regulated, and the up-regulation was abolished by the deletion of *saeRS* [89]. This study strongly suggests that WalRK can positively affect the SaeRS TCS, although the molecular mechanism is unknown.

### 5.3. SigB

The alternative sigma factor σ^B^ is a global regulator controlling the expression of genes in response to various stresses such as heat, infection, oxidative molecules and antibiotics [90,91,92]. σ^B^ was reported to activate the expression of *coa* [93,94], which has the σ^B^ promoter sequence [95]. However, σ^B^ is also known to downregulate the transcription of the *sae* operon from the P1 promoter and other Sae target genes such as *hla, hlgABC*, *nuc*, and *splABCDEF* [93]. Since sigma factors bring RNA polymerase to promoter sequences, the downregulation of the Sae-regulon by σ^B^ must be indirect, possibly mediated by regulatory proteins or small non-coding RNAs that are positively regulated by σ^B^ [96]. Indeed, in their study on the role of Sae and σ^B^ in virulence gene expression during device-related infection, Goerke et al. found no evidence of cross talk between the two regulators [97], indicating that Sae and σ^B^ are independent regulatory systems.

### 5.4. Fur

As a global regulator of iron homeostasis, Fur controls the expression of genes required for iron uptake functions in response to iron-availability [98,99,100]. In *S. aureus*, Fur also controls the genes required for biofilm formation and anti-oxidative stress response [101,102,103]. Addition of exogenous iron in the media significantly decreased the *saeRS* transcription from the constitutive P3 promoter and marginally reduced the transcription from the inducible P1 promoter [79]. In addition, the transcription of *sae* and many of the Sae-regulated genes was reduced in the *fur* mutant [79], implying that Fur may act as a positive regulator of the SaeRS TCS. However, the *sae* operon does not have Fur-binding sites, and Cho et al. has reported that *sae*P1 promoter activity was not affected by Fur [42]. Therefore, any Fur-mediated regulation of the Sae system is questionable.

### 5.5. Other Regulators

Rot (repressor of toxins) is a member of the SarA protein family and represses the expression of *hla* [20,104]. Rot is also known to reduce the promoter activity of the *sae*P1 promoter by an unknown mechanism [20]. Based on the result, Rot was suggested to repress the *hla* expression via Sae [20]. However, since the P1 promoter has no role in regulation of the transcription of Sae targets [18], it is unlikely that the *hla* repression by Rot was mediated by Sae.

Recently, it has been reported that mutation of genes encoding fatty acid kinase (*fakA* and *fakB1*/*fakB2*) significantly decreased α-hemolysin production and lowered the expression of Sae target genes [105], raising the possibility that the Fak system positively affects the SaeRS TCS. It was suggested that, similar to acetyl phosphate in Gram-negative bacteria [106], the acyl-PO_4_ form of FakB might serve as a phosphoryl group donor for SaeR [105].

## 6. The Role of Sae in the Virulence of *S. aureus*

### 6.1. Biofilm Formation

*S. aureus* forms a biofilm, which enables the bacterium to resist antimicrobial therapy and host defenses [107]. A well-defined mechanism of biofilm formation is the production of the *icaADBC*-encoded polysaccharide intercellular adhesin (PIA, also known as polymeric N-acetyl-glucosamine, PNAG) [108,109,110]. *S. aureus* also forms a biofilm in a PIA/PNAG-independent manner, utilizing diverse molecules such as surface proteins, secreted proteins, and released extracellular DNA (eDNA) [111]. Staphylococcal biofilm formation is affected by growth conditions (e.g., NaCl, glucose, human plasma etc.) [112,113] and is controlled by multiple global regulators such as SarA, Agr, SigB, and Sae. [111,113,114,115,116,117]. The Sae-regulon includes both the factors promoting biofilm formation (i.e., Coa, Emp, Eap, FnBPA, FnBPB, Hla, Hlb) [69,113,118,119,120,121] and biofilm dispersal factors (nuclease and proteases) (Table 1). Therefore, it is possible that, depending on growth conditions and strain backgrounds, the Sae system could affect biofilm formation either positively or negatively. For example, Cue et al. showed that the high kinase activity of SaeS^P^ is responsible for the poor biofilm formation of the strain Newman [117]. Indeed, either deletion of the *sae* operon or replacement of SaeS^P^ with SaeS^L^ greatly enhanced the biofilm formation. The study also indicated that the strain Newman secretes a heat-stable protein that inhibits biofilm formation. However, the nature of the biofilm inhibitor has not been determined [117]. In iron (Fe)-free cell culture medium (RPMI-1640) containing plasma, however, the Sae system was required for biofilm formation, where coagulase (Coa)-catalyzed conversion of fibrinogen into fibrin played an essential role [113]. Intriguingly, in tryptic soy broth (TSB), the Sae system was dispensable for biofilm formation [113]. Johnson et al. reported that an Fe-restricted growth condition induces biofilm formation by the strain Newman via the expression of the Sae-regulated proteins Emp and Eap (Table 1) [69]. Since Fe represses the Sae system [42], it is likely that the low iron-condition increased the overall Sae-activity, leading to the enhanced expression of Emp and Eap. Baker et al. also reported that a high level of Cu repressed the biofilm formation of the strain Newman in CRPMI medium, while Mn, Mg, and Ca did not [54]. These results are consistent with the report that the kinase activity of SaeS is inhibited by Cu, but not by Mn, Mg, or Ca [42]. In *S. aureus*, SarA plays a positive role in biofilm formation, and *sarA* mutation generally reduces staphylococcal biofilm formation [122,123]. However, *sarA* mutation does not affect biofilm formation in the strain Newman [122,124]. Intriguingly, either deletion of the *sae* operon or the replacement of SaeS^P^ with the wild type SaeS restored the positive role of SarA [124], showing that the high kinase activity of SaeS^P^ is responsible for the lack of biofilm phenotype of the *sarA* mutation in the strain Newman.

### 6.2. Invasion of Host

SCIN (staphylococcal complement inhibitor) and CHIPS (chemotaxis inhibitory protein of staphylococci) are efficient inhibitors of neutrophil chemotaxis, phagocytosis and killing [70], and the expression of both molecules are positively regulated by the SaeRS TCS (Table 1). Neutrophils, the key effectors of the host innate immune response, can release DNA in the form of neutrophil extracellular traps (NETs), which capture and eliminate microbes [125]. *S. aureus* can destroy NETs by producing nuclease, a member of the Sae-regulon (Table 1) [126]. Furthermore, *S. aureus* is known to survive within phagocytes and many types of cells, and the Sae-regulated toxins (LukAB and LukED) can damage phagocytes such as macrophages and neutrophils, helping *S. aureus* escape from the killing by the phagocytes [127]. The SaeRS TCS is required for adhering to and invading lung epithelial cells (A549) by regulating a novel hypothetical protein (SA1000) and *efb* [65]. α-hemolysin (Hla) can cause a significant damage to the plasma membrane of the human CD14^+^ monocyte [128]. The SaeRS TCS has been reported to induce the production of localized and systemic pro-inflammatory cytokines, including tumor necrosis factor alpha (TNF-α), Interferon gamma (IFN-γ), Interleukin (IL) 6 and IL-2 [40,42,129].

Considering the protective roles of the SaeRS TCS described above, it is not surprising that the SaeRS TCS is required for disease progression in several animal models of infection including silkworm and nematode killing [130,131], murine survival and skin infection [132], infective endocarditis [4], necrotizing pneumonia and skin infection [133], osteomyelitis [74], and sepsis [134]. The SaeRS TCS was even needed for staphylococcal gastrointestinal dissemination and transmission following bacteremia [135].

## 7. Conclusions

In *S. aureus*, the SaeRS TCS plays a key role in production of over 20 virulence factors (Table 1), and pathogenesis of the bacterium. None of those virulence genes are found in the closely related *S. epidermidis*, which also has the SaeRS TCS [136,137]. Therefore, *S. aureus* seems to have evolved its virulence capacity by acquiring those virulence genes and placing them under the control of the SaeRS TCS. Despite intense research on the Sae system over the past two decades, we still do not know how cognate Sae signals such as HNPs are sensed by SaeS. Do HNPs directly bind SaeS? Or do they activate SaeS via a hitherto-unidentified receptor molecule? On top of that, HNPs do not activate the SaeRS TCS in certain strain backgrounds, and the reason is completely unknown. As discussed in Section 3, the SaeRS TCS is affected by diverse molecules such as HNPs, LTA, calprotectin, and metal ions. This might be due to the fact that the entire transmembrane domain of SaeS is either buried in the membrane (TMs) or in close contact (the linker peptide) with molecules in the membrane. Since the kinase activity of SaeS appears to respond sensitively to the overall conformation of the transmembrane domain, any molecules, either exogenous or endogenous, that alter the conformation of the SaeS transmembrane domain are expected to serve as a legitimate signal for the SaeRS TCS. Certainly, more biochemical and mechanistic studies are needed to define the mechanism by which the enzymatic activity of SaeS is modulated by those signals. Finally, there is no direct evidence yet that the SaeRS TCS is directly regulated by other regulatory systems. In particular, the SaeRS TCS and the Agr quorum sensing system are likely independent of one another.

## Figures and Tables

**Table 1:** Direct targets of the SaeRS TCS.

Locus ID ^1^	Name	Product	SaeR Binding Sequence	Evidence ^2^	References ^3^
**NWMN_0166**	*coa*	coagulase	**GTTAA**TGCTTT**GTTTA** **GTTAA**TATATAG**TTAA**	EMSA, MA, NB, RA	[21,48,63,64,65]
**NWMN_0388**	*ssl1nm*	enterotoxin-like toxin	**GTTAA**ATGAGG**TTTAA**	2D	[66]
**NWMN_0389**	*ssl2nm*	enterotoxin-like toxin	**GTTAA**AAACAG**GTTAA**	2D	[66]
**NWMN_0390**	*ssl3nm*	enterotoxin-like toxin	**GTTAA**AAGGGG**TTTAA**		
**NWMN_0391**	*ssl4nm*	enterotoxin-like toxin	**GTTAA**ACAAGG**TTTAA**		
**NWMN_0392**	*ssl5nm*	enterotoxin-like toxin	**ATTAA**ACATGG**TTTAA**	MA, RT	[65,67]
**NWMN_0393**	*ssl6nm*	enterotoxin-like toxin	**GTTCA**AAAATA**GTTAA** **GTTAA**AAAGAG**GTTAA**		
**NWMN_0394**	*ssl7nm*	enterotoxin-like toxin	**GTTCA**AAAATA**GTTAA** **GTTAA**AAAGAG**GTTAA**	EMSA, 2D, WB	[39,66]
**NWMN_0395**	*ssl8nm*	enterotoxin-like toxin	**GTTAA**TGAAGA**GCTAA** **CTTAA**ATCATT**GTTAA** **ATTAA**ACGAGT**GTTAA**	RT	[67]
**NWMN_0396**	*ssl9nm*	enterotoxin-like toxin	**ATTAA**AAATCA**GTTAA**	EMSA, WB	[39]
**NWMN_0400**	*ss11nm*	enterotoxin-like toxin	**ATTAA**TTTTTA**GTTAA**	EMSA, 2D, WB	[39,66]
**NWMN_0677**	*saeP*	SaeP protein	**GTTAA**GCGATA**TTTAA** **GTTAA**GAATTA**GTTAA**	EMSA, MA, NB, RA	[15,19,48]
**NWMN_0758**	*ssr/emp*	extracellular matrix binding protein	**GTTAA**GACAAC**GTTTA** **GTTTA**CTTCAA**GTTAA**	1D, MA, RA	[48,68,69]
**NWMN_0760**	*nuc*	nuclease	**ATTAA**ATTTTT**ATTAA**	EMSA, 2D, MA, RA,WB	[33,48,66]
**NWMN_1066**		fibrinogen-binding related protein	**ATTAA**TGTTTA**GTTAA** **GTTAA**TAAATA**GTTAA**	MA	[48,65]
**NWMN_1069**	*efb*	similar to fibrinogen binding protein	**ATTAA**TAATTA**GTTAA**	MA	[48,65]
**NWMN_1073**	*hla*	α-hemolysin	**GTTAA**TATATA**GTTAA**	EMSA, MA,RA	[21,48,64,65]
**NWMN_1706**	*splA*	serine protease	**TTTAA**TAAAAC**GTTAA** **GTTAA**TTAATA**TTTAA**	2D	[66]
**NWMN_1708**		homologous to ear	**GTTAA**TAGATA**GTTAA** **GTTAA**TACATT**TTTGA**		
**NWMN_1719**	*lukE*	leukocidin LukE	**TTTAA**TGAACA**GTTAA** **GTTAA**TAATCA**GTTAA**		[27]
**NWMN_1872**	*map/eap*	MHC class II analog protein	**ATTAA**TATTCA**GTTAA**	1D, NB, RA	[27,32,68,69]
**NWMN_1873**	*hlb*	β-hemolysin (truncated)	**ATTAA**CTGAAT**ATTAA**	MA,NB	[12,21,65]
**NWMN_1876**	*scn*	staphylococcal complement inhibitor	**GTTAA**TGAATA**ATTAA**	RA	[70]
**NWMN_1877**	*chp*	chemotaxis-inhibiting protein	**TTTAA**TTTTTA**GTTAA** **ATTAA**TTTCAA**GTTAA**	1D, MA,RA	[27,48,70]
**NWMN_1928**	*lukA* (*lukG*)^4^	leukocidin LukA (LukG)	**TTTAA**TAAATA**GTTAA**	1D, MA	[27,65,71,72]
**NWMN_2317**	*sbi*	IgG binding protein	**GTTAA**TAATTA**GTTAA**	1D, MA	[21,48]
**NWMN_2319**	*hlgC*	γ-hemolysin component C	**GTTAA**TGAACA**GTTAA**	1D, MA	[27,48,65]
**NWMN_2397**	*fnbB*	fibronectin binding protein B	**GTTAA**TAAAAA**GTTAA**	MA	[48,65]
**NWMN_2399**	*fnbA*	fibronectin binding protein A	**GTTAA**TGAAAA**GTTAA** **ATTAA**TTTTAT**GTTAA**	NB,WB	[19,21]
**SA1819**	*tst*	toxic shock syndrome toxin	**ATTAA**TATATA**TTTAA** **ATTTA**GAGATG**GTTAA**	EMSA,1D,RA,RT	[73]
**Possible targets**					
**NMTN_0095**	*capA*	capsular polysaccharide synthesis enzyme	**GTTTA**AAAGTA**ATTAA**		
**NWMN_0157**		conserved hypothetical protein	**ATTAA**TAAATA**GTTAA**		-
**NWMN_0362**		hypothetical protein	**GTTAA**TCAAGA**GTTAA** **GTTAA**GATGAA**TTTAA**		-
**NWMN_0403**	*lpl1nm*	lipoprotein	**TTTAA**TAAATA**GTTAA**		-
**NWMN_1533**	*his*	histidyl-t-RNA synthetase	**GTTAA**ACGTAC**GTTAA**		-
**NWMN_1880**	*sak* ^5^	staphylokinase	**GTTAA**ATATTT**GTTAA** **GTTAA**TTATTT**TTTAA**		[12,70]
**NWMN_2536**	*aur*	zinc metalloproteinase aureolysin	**TTTAA**AATATA**ATTAA**		[24,66,74].
**NWMN_2592**		2-oxoglutarate/malate translocator	**GTTAA**CAACAC**GTTAA**		-

^1^ Based on the genome of the strain Newman except for *tst*, which is from the strain N315; ^2^ 1D, SDS-PAGE; 2D, 2D gel electrophoresis; EMSA, Electrophoretic mobility shift assay; MA, Microarray analysis; NB, Northern blot; RA, Reporter assay; RT, RT-PCR; WB, Western blot; ^3^ Only select references are shown; ^4^ Although NWMN_1928 is annotated as *lukS* in Newman genome sequence, it was renamed to *lukA* (*lukG*) [71,72]; ^5^ Although *sak* contains a perfect SBS, according to the references, the transcription of *sak* is not significantly affected by *sae* mutation.

## Abbreviations

The following abbreviations are used in this manuscript:

TCS	Two-component system
SBS	SaeR binding sequence
HK	Histidine kinase
SaeR^DB^	The DNA binding domain of SaeR
LTA	Lipoteichoic acid
